# Optimal care and design of the tracheal cuff in the critically ill patient

**DOI:** 10.1186/2110-5820-4-7

**Published:** 2014-02-27

**Authors:** Emmanuelle Jaillette, Ignacio Martin-Loeches, Antonio Artigas, Saad Nseir

**Affiliations:** 1 Pôle de Réanimation, Hôpital Salengro, CHRU de Lille, Université Nord de France, Lille, France; 2 Critical Care Center, Corporacion Sanitaria Universitaria Parc Tauli, Sabadell University Hospital, Universidad Autonoma de Barcelona, CIBER Enfemedades Respiratorias, Sabadell, Spain

**Keywords:** Intubation, Complications, Microaspiration, Ventilator-associated pneumonia, Tracheal ischemia

## Abstract

Despite the increasing use of non-invasive ventilation and high-flow nasal-oxygen therapy, intubation is still performed in a large proportion of critically ill patients. The aim of this narrative review is to discuss recent data on long-term intubation-related complications, such as microaspiration, and tracheal ischemic lesions. These complications are common in critically ill patients, and are associated with substantial morbidity and mortality. Recent data suggest beneficial effects of tapered cuffed tracheal tubes in reducing aspiration. However, clinical data are needed in critically ill patients to confirm this hypothesis. Polyurethane-cuffed tracheal tubes and continuous control of cuff pressure could be beneficial in preventing microaspiration and ventilator-associated pneumonia (VAP). However, large multicenter studies are needed before recommending their routine use. Cuff pressure should be maintained between 20 and 30 cmH_2_O to prevent intubation-related complications. Tracheal ischemia could be prevented by manual or continuous control of cuff pressure.

## Introduction

Nowadays, tracheal intubation is less performed in the ICU because of the increasing use of non-invasive ventilation and high-flow nasal-oxygen therapy [1-4]. However, this invasive procedure is still performed in a high percentage of critically ill patients requiring mechanical ventilation. A large multicenter epidemiological study was performed in 927 ICUs, and included 18,302 patients requiring mechanical ventilation for > 12 hours [5]. The percentage of patients requiring invasive mechanical ventilation through a tracheal tube decreased from 95% in 1998 to 86% in 2010. However, 30% of patients who received non-invasive ventilation required subsequent intubation.

In this narrative review, we will report and discuss data on long-term intubation-related complications. We will also review the impact of shape, volume, and material of the tracheal cuff on the incidence of these complications. Finally, we will discuss data on the optimal care for the tracheal cuff.

Data for this review were identified through searches of PubMed, and from bibliographies of relevant articles. We performed a comprehensive search in PubMed, from 1983, through 2013, using the terms “tracheal cuff AND microaspiration”, “tracheal cuff AND pneumonia”, “tracheal cuff AND ischemia”, “tracheal cuff AND complications”. The search was limited to publications in English and French.

## Review

### Complications related to the tracheal cuff

Complications related to intubation could be classified into immediate and long-term complications. Microaspiration and tracheal ischemia are common long-term complications in the critically ill patient.

### Microaspiration

**Figure 1:**
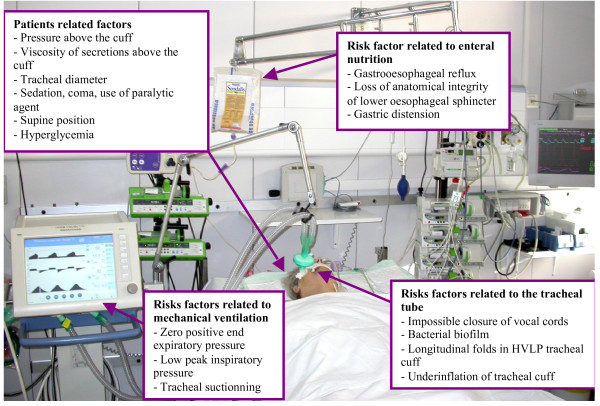
Risk factors for microaspiration in the critically ill patient.

Microaspiration of contaminated oropharyngeal and gastric secretions is the major route of entry for bacteria into the lower respiratory tract [6]. Tracheobronchial colonization might progress to ventilator associated tracheobronchitis and pneumonia when local and general host defenses are altered, and when the quantity and virulence of bacteria are high [7]. Microaspiration occurs in up to 88% of intubated critically ill patients [8]. Several factors are implicated in the pathogenesis of microaspiration, including tracheal tube, mechanical ventilation, enteral nutrition, and patient-related factors [6] (Figure 1). Therefore, prevention of microaspiration and ventilator-associated pneumonia (VAP) is a multifactorial process that should take into account all of these factors [9].

The tracheal tube prevents closure of vocal cords, and represents an access for bacteria to progress in the trachea through the folds of high-volume low-pressure tracheal tubes into the lower respiratory tract [7]. Underinflation of the tracheal cuff under 20 cmH_2_O results in microaspiration, and was identified as an independent risk factor for VAP [10]. However, this cut-off is based on one single center study including few patients. Therefore, microaspiration could probably occur at highest or lowest levels of cuff pressure. Mechanical ventilation plays an important role in microaspiration, and cuff pressure is tightly correlated with airway pressure [11]. The application of a positive end expiratory pressure was identified as protective from microaspiration and VAP [12,13]. Tracheal suctioning could be associated with higher risk for microaspiration, because of negative pressure applied during this procedure. It has been demonstrated that leakage rate around the cuff depends on the difference in pressure between the two areas above and below the cuff [14]. A recent study was conducted in 25 intubated critically ill patients to determine the impact of tracheal suctioning on leakage of blue dye, diagnosed using fiberoptic bronchoscopy [15]. Whilst blue dye was observed in the folds within the cuff wall in a larger number of patients before compared with after suctioning, the difference did not reach statistical significance (*P* = 0.063).

Enteral nutrition through a nasogastric tube is a recognized risk factor for microaspiration and VAP mainly because of gastroesophageal reflux [16]. Gastric distension, loss of anatomical integrity of the lower esophageal sphincter, increased frequency of transient sphincter relaxation and oropharyngeal dysphagia via desensitization of the pharyngoglottal adduction reflex are the main mechanisms explaining the role of enteral nutrition in aspiration [17].

Patient-related factors could be classified into local and general factors. Viscosity of secretions above the cuff and tracheal diameter are the main local factors influencing microaspiration [14]. Sedation, coma, supine position, and hyperglycemia are general patient-related factors also influencing microaspiration [18]. Sedation and coma are well known risk factors for microaspiration and VAP. Deglutition alteration, impairment in tubular esophageal motility and increased gastrointestinal reflux disease might explain this relationship [19]. Supine position was identified as a major risk factor for aspiration and VAP in studies using technetium 99 m [20]. Further, hyperglycemia increases the risk for microaspiration by delayed gastric emptying.

Whilst microaspiration is common in intubated critically ill patients, its diagnosis remains challenging. The gold standard for diagnosing microaspiration of gastric contents is technetium 99 m [21]. However, this marker is radioactive, and its use is not allowed in the ICU without specific precautions. Other markers such as blue dye [15], bile acids [22], pepsin [23], and amylase [24,25] were recently used. However, several limitations of these markers should be taken into account, including low specificity, qualitative assessment of microaspiration, short window for their detection in tracheal aspirates, technical difficulties, and absence of validation compared with the gold standard.

### Tracheal ischemia

Few studies have evaluated the incidence of ischemic tracheal lesions in critically ill patients [26-28]. Based on their results, the incidence of these lesions varies from 31% to 95% of critically ill patients. The most frequently described lesions are hyperemia, ischemia, ulcer, granuloma, and tracheal rupture. Usually, these lesions are seen at the zone of contact with the tracheal cuff.

**Figure 2:**
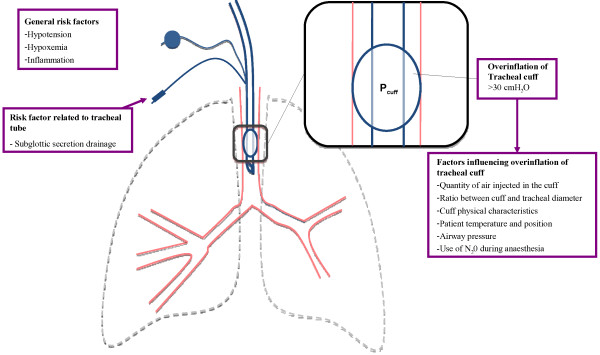
**Risk factors for ischemic tracheal lesions.** HPLV, high-volume low-pressure.

Overinflation of the tracheal cuff (> 30 cmH_2_O) is the main risk factor for ischemic tracheal lesions [29]. Similar as for microaspiration, the cut-off (30 cmH_2_O) for ischemic lesions is based on only one study, and these lesions may well occur at different levels of cuff pressure. Several factors influence cuff pressure, including quantity of air injected in the cuff [30], the ratio between cuff and tracheal diameter, cuff physical characteristics [31], patient temperature [32], airway pressure [11], and patient position [33]. Other risk factors for tracheal ischemia include hypotension [34], hypoxemia [27,35], inflammation [36], and subglottic secretion drainage [37,38] (Figure 2). Seegobin and van Hasselt [29] performed a prospective study in 40 patients intubated with a high-volume low-pressure cuffed tracheal tube for surgery under general anesthesia. They evaluated tracheal perfusion at different cuff pressure levels (from 30 to 100 cmH_2_O), and found important reduction in mucosal capillary blood flow above 30 cmH_2_O, and a total obstruction above 50 cmH_2_O. Animal studies with histological examination confirmed these results [39]. An animal study demonstrated that tracheal ischemia was substantially increased by hypotension [34]. Further, two clinical studies found hypoxemia to increase the severity of tracheal injury [27,35]. Another suggested mechanism for tracheal ischemia is inflammation resulting from the presence of the tracheal tube, and repeated ischemia-reperfusion [36]. Subglottic secretion drainage was also reported as a risk factor for tracheal ischemia. However, only one animal, and a small clinical study implicated subglottic secretion drainage in tracheal ischemic lesions [37,38]. Our group performed a study to determine risk factors for ischemic tracheal lesions in a cohort of 96 critically ill patients. These lesions were diagnosed using fiberoptic tracheoscopy performed during the 24 hours following extubation [28]. The only independent risk factor for ischemic tracheal lesions was duration of assist-control mechanical ventilation through a tracheal tube. This result could be explained by the fact that airway pressure is usually higher in assist-control compared with pressure-control mechanical ventilation, resulting in higher cuff pressure.

Several complications could occur after severe tracheal ischemic lesions, such as tracheal stenosis [40], tracheal rupture [41], tracheobronchiomalacia [42], tracheoinnominate artery fistula [43], and tracheoesophageal fistula [44]. However, few data are available on the transition from ischemic tracheal lesions to these complications. In the above-discussed study [28], we performed a second fiberoptic tracheoscopy two weeks after the last extubation in 22 patients with severe ischemic lesions. The lesions completely healed in the majority of patients, suggesting that when cuff pressure is correctly controlled, even with a manometer, no complication would occur.

### Different types of tracheal cuff

Tracheal tubes can be classified based on the relationship between volume and cuff pressure. Cuff shape and material can also differ between tracheal tubes.

### Pressure-volume relationship

The first tracheal cuffs were small-volume high-pressure. These cuffs allow sealing with a small area of contact with tracheal wall, but require high pressure (> 50 cmH_2_O) to obtain tracheal sealing. The cuff pressure allowing adequate sealing is much higher than the perfusion pressure of tracheal mucosa resulting in tracheal injury. Their use was associated with high incidence of ischemic tracheal lesion and subsequent complications such as tracheal stenosis and rupture. These cuffs are unfortunately still available on the market, and used in some patients, but should be abandoned because of the high risk for tracheal injury.

Most currently used cuffs are high-volume low-pressure. Several *in vitro* and animal studies showed that these cuffs require lower pressure to obtain sealing, and were associated with reduced ischemic tracheal lesions [39,45]. Honeybourne and colleagues [46] performed a randomized controlled study in 28 patients requiring intubation and mechanical ventilation for 24 hours. Patients were randomized to be intubated with a high-volume low-pressure or a low-volume high-pressure cuff. A fiberoptic bronchoscopy was performed at the time of extubation to evaluate ischemic lesions based on a score including edema, inflammation, and ulceration. The authors reported significantly lower incidence of ischemic tracheal lesions in patients intubated with high-volume low-pressure cuffed tubes compared with those intubated with low-volume high-pressure cuffed tubes. However, several studies have shown that even when tracheal cuff pressure is adjusted below 30 cmH_2_O, ischemic tracheal lesions could still occur in patients intubated with high-volume low-pressure cuffs [47,48]. One potential explanation is that manual control of cuff pressure is not accurate. In addition, the target of < 30 cmH_2_O might be inappropriate in critically ill patients, since these lesions could be seen at lowest cuff pressure level, especially in patients with shock or hypoxemia. To obtain optimal sealing with high-volume low-pressure cuffs, they should be inflated to obtain a volume above tracheal diameter. As a consequence, channels occur between the polyvinylchloride (PVC) cuff and tracheal wall, resulting in microaspiration of contaminated secretions above the cuff into the lower respiratory tract.

Recently, low-volume low-pressure cuffs have been studied in critically ill patients. These silicone cuffs provide accurate sealing of the trachea with acceptable pressure (< 30 CmH_2_O). Young and colleagues [49] evaluated these cuffs *in vitro*, in patients anesthetized for surgery, and in critically ill patients. Their results showed significantly lower leakage using these cuffs compared with conventional high-volume low pressure PVC-cuffed tracheal tubes. However, in their clinical studies two additional measures for prevention of aspiration were used, namely, continuous control of cuff pressure and subglottic secretion drainage. Therefore, it is difficult to determine whether the reduced aspiration rate found in their study was related to the low-volume low-pressure cuff or to other interventions. A subsequent retrospective study from the same group reported zero VAP rate in a cohort of 53 patients, in spite of reintubation of 83% study patients at ICU admission, suggesting a benefit from these tracheal tubes [50].

### Cuff shape

Three cuff shapes are available in barrel (standard), cylindrical, and conical (tapered) shape. Unfortunately, few data are available on the impact of cuff shape on microaspiration, and VAP. Results of *in vitro* studies suggest improved sealing using the conical-shaped cuffs [51-53]. This beneficial effect is attributed to the fact that tracheal diameter is not constant, and that a conical cuff might provide a sealing zone whatever the tracheal diameter. A recent randomized study found that short-term use of taper-shaped PVC cuffs in surgical patients resulted in more effective sealing of the tracheal lumen in comparison with traditional barrel-shaped PVC cuffs [54]. However, further large randomized studies are needed to evaluate the impact of cuff shape on microaspiration and VAP in critically ill patients.

### Cuff material

Polyurethane could reduce the risk of microaspiration and VAP. This material is much thinner than PVC, resulting in reduced formation of folds between the cuff and tracheal wall. Several *in vitro* studies showed that polyurethane significantly reduced leakage around the tracheal cuff [51,52]. However, results of clinical studies are controversial. Lucangelo and colleagues [55] performed a randomized controlled study to evaluate the effect of polyurethane and a positive end expiratory pressure on microaspiration of blue dye, diagnosed using fiberoptic bronchoscopy, in 40 critically ill patients. These combined measures were found to significantly reduce microaspiration. In a before-after study, performed in 76 consecutive patients and using pepsin as a marker of microaspiration, our group also found polyurethane to be associated with significantly less microaspiration [56]. In contrast, a recent randomized controlled study using radioactive technetium m99 to diagnose microaspiration, found no significant difference in microaspiration between patients intubated with polyurethane-cuffed compared with those intubated with PVC-cuffed tracheal tubes [57].

Three clinical studies evaluated the impact of polyurethane on the incidence of hospital acquired pneumonia [58-60]. All these studies found significantly lower incidence of VAP or nosocomial pneumonia. However, some limitations of these studies should be taken into account, including the combined use of subglottic secretion drainage [59], before-after design [58], and clinical diagnosis of pneumonia [58,60].

### Regulation of cuff pressure

Based on recent recommendations, cuff pressure should be checked and adjusted around 25 cmH_2_O, at least twice a day using a manometer. Unfortunately, this recommendation is not followed in a large number of ICUs.

### Discontinuous manual regulation

Even when well applied, discontinuous control of cuff pressure using a manometer is not efficient in keeping cuff pressure within the recommended range (20 to 30 cmH_2_O). In a cohort of 101 critically ill patients intubated with a PVC-cuffed tube, cuff pressure was continuously recorded for eight hours after manual adjustment of cuff pressure at 25 cmH_2_O [61]. Only 18% of study patients spent 100% of recording time with normal (20 to 30 cmH_2_O) cuff pressure. Fifty-four per cent of study patients developed cuff underinflation, 73% developed cuff overinflation, and 44% developed both. Thirty-three per cent of study patients developed underinflation or overinflation for > 30 minutes. No modifiable risk factor for underinflation or overinflation of the tracheal cuff was identified in that study. Duration of intubation and absence of sedation were independently associated with underinflation of the tracheal cuff. Percentage of time spent with underinflation of the tracheal cuff significantly increased during the recording period, suggesting that cuff pressure should be checked and regulated more frequently using a manometer. However, previous studies showed that the connection of manometer to external cuff is associated with a sudden drop in cuff pressure which might result in microaspiration [62,63]. In another prospective study, our group aimed to determine the impact of cuff shape and material on cuff pressure [56]. Patients were intubated with PVC standard (n = 26), polyurethane cylindrical (n = 22), or polyurethane conical-shaped cuffed (n = 28) tracheal tubes. Cuff pressure was continuously recoded for 24 hours, and adjusted by nurses using a manometer thrice a day. Patients spent 51% to 62% of recording time within the normal range. No significant difference was found in percentage of time spent with underinflation, and overinflation between the three groups. However, coefficient of variation of cuff pressure was significantly higher in patients intubated with conical-shaped cuffed tracheal tubes compared with the two other groups.

Whilst control of cuff pressure using a manometer is not efficient in keeping cuff pressure within the zone of 20 to 30 cmH_2_O, this measure is probably better than no control at all. Several studies have shown that the finger method is inaccurate in estimating cuff pressure, and that patients could spend a long period of time with underinflation or overinflation of cuff pressure when no adjustment is performed at all [30]. Further, a recent randomized controlled study performed on a large number of patients scheduled for elective surgery found proper control of cuff pressure using a manometer to be associated with significantly reduced clinical complications such as cough, sore throat, hoarseness, and blood-streaked expectorant, related to cuff overinflation compared with no measurement of cuff pressure [64]. However, to our knowledge, no study has evaluated the impact of discontinuous control of cuff pressure using a manometer on the incidence of complications related to underinflation or overinflation of tracheal cuff in ICU patients.

In order to improve the efficiency of manual control of cuff pressure using a manometer, an alarm could be used to accelerate nurse intervention, and to reduce time spent with tracheal cuff underinflation or overinflation. Sole and colleagues [65] performed a randomized controlled cross-over study to evaluate the effect of continuous monitoring of cuff pressure, combined with an alarm, in maintaining cuff pressure in the target range. Patients (n = 32) received two 12-hour periods of routine care and continuous monitoring of cuff pressure. During the intervention period, when pressure fell out of the target range of 20 to 30 cmH_2_O, an alarm warned the nurse, who adjusted cuff pressure. This intervention allowed reduction of the percentage of cuff pressure values out of range (11.1% versus 51.7%, *P* < 0.001). However, the effect of this intervention on VAP prevention is unknown. Moreover, this procedure, adding extra work for nurses and an extra alarm in today’s already noisy and stressful ICU environment for patients and healthcare workers, could hardly be an effective solution for maintenance of cuff pressure within therapeutic range.

### Continuous regulation

Recently, several devices aiming at continuously controlling cuff pressure were studied. Two different types of devices are available, pneumatic and electronic. The efficiency of the pneumatic device continuously controlling cuff pressure was demonstrated in two studies performed on critically ill patients and in animals intubated with PVC-cuffed tracheal tubes [66,67]. These data were recently confirmed by a randomized study performed by our group on patients intubated with polyurethane-cuffed tracheal tubes [68]. Whilst efficiency of electronic devices was reported by an *in vitro* study, few clinical data are available on the efficiency of electronic devices in controlling cuff pressure. In a recent randomized cross-over study, Brisson and colleagues [69] compared the efficiency of an electronic device to that of a pneumatic one in ten critically ill patients. Cuff pressure was continuously recorded for nine hours (three hours with routine care using a manometer, three hours with continuous control using an electronic device (Tracoe™) and three hours of continuous control of cuff pressure using a pneumatic device). The authors found underinflation of P_cuff_ to be more frequent using the electronic device compared with the pneumatic device (8% versus 0%, respectively), and attributed this result to the over compensation for any elevated cuff pressure.

The impact of pneumatic and electronic devices on continuous control of cuff pressure is an important issue. However, only clinical outcomes such as reduction of microaspiration, VAP, and tracheal ischemia could be used to justify the extra cost generated from their use. Unfortunately, few clinical studies were performed to determine the impact of continuous control of cuff pressure on long-term intubation-related complications.

Our group performed a randomized controlled animal study to determine the impact of continuous control of cuff pressure on ischemic tracheal lesions [66]. Piglets were intubated with a PVC-cuffed tracheal tube, and ventilated for 48 hours under sedation and neuromuscular blocking agents. They were randomized to receive continuous control of cuff pressure using a pneumatic device (n = 6) or routine care using a manometer (n = 6). Overinflation of the tracheal cuff was performed in the two groups in order to mimic overinflation periods in critically ill patients. Animals were sacrificed after 48 hours of mechanical ventilation, and the physician who performed histological examination of the piglets’ trachea was blinded to study group assignment. Whilst the device was efficient in continuously controlling cuff pressure, no significant difference was found in macroscopic or microscopic lesions found in study animals. One potential explanation for the absence of significant effect of continuous control of cuff pressure on ischemic tracheal lesions is the short duration of intubation.

Two randomized controlled single center clinical studies evaluated the impact of continuous control of cuff pressure on the incidence of intubation-related complications [23,70]. Valencia *et al*. [70] performed a randomized study in 142 critically ill patients without pneumonia or aspiration at ICU admission. Patients received continuous control of P_cuff_ using an electronic artisanal device previously validated by the same authors (intervention group, n = 73) or routine care of P_cuff_ (control group, n = 69). Underinflation of tracheal cuff was significantly less frequent in intervention compared with control group (45.3% versus 0.7%, *P* < 0.001). However, no significant difference was found in the incidence of microbiologically confirmed VAP between the two groups (15% in the two groups). Similarly, no significant difference was found in the incidence of suspected VAP (22% versus 29%), distribution of early and late-onset VAP, causative microorganisms, ICU (27% versus 23%) or hospital (41% versus 33%) mortality. Some limitations of this study should be taken into account including single center design, absence of blinding, exclusion of patients with suspected pneumonia at ICU admission, and absence of evaluation of microaspiration, colonization, or tracheal ischemia.

Our group performed a randomized controlled study to determine the impact of continuous control of cuff pressure on microaspiration of gastric contents [23]. The secondary objectives were the impact of this intervention on tracheobronchial colonization, VAP incidence, and ischemic tracheal lesions. Patients requiring mechanical ventilation through a PVC-cuffed tracheal tube for > 48 hours were eligible, and were randomized to receive continuous control of cuff pressure using a pneumatic device (intervention group, n = 61) or routine care (control group, n = 61). Target cuff pressure was 25 cmH_2_O in the two groups. Pepsin was quantitatively measured in all tracheal aspirates during the 48 hours following randomization. Abundant microaspiration was defined as the presence of pepsin at significant level (> 200 ng/mL) in > 65% of tracheal aspirates. Quantitative tracheal aspirate was performed at intubation, and thrice a week. The pneumatic device was efficient in controlling cuff pressure. Pepsin was measured in 1,205 tracheal aspirates. Percentage of patients with abundant microaspiration (18% versus 46%, *P* = 0.002, OR (95% CI) 0.25 (0.11 to 0.59)), bacterial concentration in tracheal aspirates (mean ± SD 1.6 ± 2.4 versus 3.1 ± 3.7 log_10_ cfu/ml, *P* = 0.014), and VAP rate (9.8% versus 26.2%, *P* = 0.032, 0.30 (0.11 to 0.84)) were significantly lower in the intervention group compared with the control group. Further, percentage of days in the ICU with antimicrobials was significantly lower in the intervention group compared with the control group (median (Interquartile range) 83 (56, 100) versus 100 (75, 100), *P* = 0.049. However, no significant difference was found in tracheal ischemia score (4.5 (1 to 6) versus 4.5 (1 to 7), *P* = 0.9) between the two groups. Several limitations of this study should also be outlined, including the fact that pepsin was only measured during the 48 hours following randomization, single center design, absence of blinding, and important proportion of study patients who had pneumonia at ICU admission. The absence of significant difference in tracheal ischemia score between the two groups could be explained by the fact that this study was not powered to detect such an effect. In addition, routine care was optimal in control group. Further, previous studies showed that overinflation of the tracheal cuff was not permanent and occurred during cough, patient mobilization and patient-ventilator asynchrony [71]. A previous study showed that overinflation of the tracheal cuff for up to 30 minutes was associated with significantly reduced but completely reversible ischemia [29]. Therefore, short overinflation of the tracheal cuff might not be harmful in critically ill patients, and continuous control of P_cuff_ would not add much in this scenario. Finally, another explanation could be the higher P_cuff_ in patients receiving continuous control of P_cuff_ compared with those receiving routine care, even if the median P_cuff_ is < 30 cmH_2_O. A randomized controlled animal study found ischemic tracheal lesions in animals intubated and ventilated for 48 hours despite continuous control of P_cuff_ (< 30 cmH_2_O) [67]. Further randomized controlled studies are needed to determine the impact of continuous control of P_cuff_ on ischemic tracheal lesions.

Several differences between these two studies might explain the different results on VAP prevention [72]. Whilst VAP incidence was the primary outcome in Valencia’s study, it was a secondary outcome in ours. Patient population was also different between the two studies, with more surgical patients (28% versus 0%), and patients with respiratory disorders (38% versus 27%) in Valencia’s study than in ours. In addition, the rate of microbiologically confirmed VAP was lower in Valencia’s study compared with ours (15% versus 26%). However, the most important difference between these studies is probably the different devices used to control P_cuff_. Although Valencia *et al*. used an electronic device, we used a pneumatic device to continuously control P_cuff_. As discussed previously, the pneumatic device is probably more efficient in controlling P_cuff_. Further, the percentage of P_cuff_ determinations in the normal range (20 to 30 cmH_2_O) was lower in Valencia’s study compared with ours (79% versus 98%).

## Conclusions

Microaspiration and ischemic tracheal lesions are common intubation-related complications. Prevention of these complications should take into account all pathophysiologic factors. Cuff pressure should be maintained between 20 to 30 cmH_2_O, if possible using a device allowing continuous control. The polyurethane-cuffed tracheal tubes could be an interesting measure to prevent microaspiration and pneumonia. Clinical data are lacking to support the use of the tapered tracheal cuff to prevent microaspiration in critically ill patient. Further studies should determine the impact of continuous control of cuff pressure on the incidence of intubation-related complications, and evaluate the impact of cuff material and shape on microaspiration and VAP.

## Abbreviations

ICU: Intensive care unit; PVC: polyvinyl chloride; VAP: ventilator associated pneumonia.

## Competing interests

SN: Covidien (lecture); Other authors: none.

## Authors’ contributions

EJ and SN performed the literature search. EJ, IM, AA, and SN drafted the manuscript. All authors read and approved the final version.
